# NEDD4 attenuates phosgene‐induced acute lung injury through the inhibition of Notch1 activation

**DOI:** 10.1111/jcmm.17296

**Published:** 2022-03-31

**Authors:** Yiru Shao, Zhifeng Jiang, Daikun He, Jie Shen

**Affiliations:** ^1^ Center of Emergency & Intensive Care Unit Jinshan Hospital Fudan University Shanghai China; ^2^ Key Laboratory of Chemical Injury, Emergency and Critical Medicine of Shanghai Municipal Health Commission Shanghai China; ^3^ Medical Research Center for Chemical Injury, Emergency and Critical Care of Chemical Injury Jinshan Hospital Fudan University Shanghai China

**Keywords:** NEDD4, Notch1, phosgene, pulmonary inflammation

## Abstract

Phosgene gas leakage can cause life‐threatening acute lung injury (ALI), which is characterized by inflammation, increased vascular permeability, pulmonary oedema and oxidative stress. Although the downregulation of neuronal precursor cell‐expressed developmentally downregulated 4 (NEDD4) is known to be associated with inflammation and oxidative damage, its functions in phosgene‐induced ALI remain unclear. In this study, rats with phosgene‐induced ALI were intravenously injected with NEDD4‐overexpressing lentiviruses to determine the functions of NEDD4 in this inflammatory condition. NEDD4 expression was decreased in the lung parenchyma of phosgene‐exposed control rats, whereas its expression level was high in the NEDD4‐overexpressing rats. Phosgene exposure increased the wet‐to‐dry lung weight ratio, but NEDD4 abrogated this effect. NEDD4 overexpression attenuated phosgene‐induced lung inflammation, lowering the high lung injury score (based on total protein, inflammatory cells and inflammatory factors in bronchoalveolar lavage fluid) and also reduced phosgene‐induced oxidative stress and cell apoptosis. Finally, NEDD4 was found to interact with Notch1, enhancing its ubiquitination and thereby its degradation, thus attenuating the inflammatory responses to ALI. Therefore, we demonstrated that NEDD4 plays a protective role in alleviating phosgene‐induced ALI, suggesting that enhancing the effect of NEDD4 may be a new approach for treating phosgene‐induced ALI.

## 1 INTRODUCTION

Phosgene (carbonyl chloride, COCl_2_) is a noxious and asphyxiating gas that induces acute lung injury (ALI) or acute respiratory distress syndrome.^1^ Previously used as a chemical counterinsurgency agent during World War I, it is nowadays applied as an essential and indispensable large‐stock bulk intermediate for the synthesis of plastics and other building materials.^1^ The intentional release or accidental discharge of this gas in a public area would lead to multiple fatalities. Phosgene‐induced ALI is characterized by acute noncardiogenic pulmonary oedema associated with an influx of neutrophils into the lungs.^2^ Specifically, the gas causes damage to lung epithelial cells and elicits an inflammatory response.^3^ Despite the high probability of death that can ensue from accidental exposure to phosgene, there are currently no specific options available for its treatment.

Neuronal precursor cell‐expressed developmentally downregulated 4 (NEDD4) belongs to a family of HECT‐type E3 ubiquitin ligases whose members are homologous to the C terminus of E6‐associated protein (E6‐AP), an E3 ligase that helps to degrade some proteins through ubiquitination and endocytosis.^4^ In humans, this family contains nine ligases with the following protein symbol designations: NEDD4, NEDD4L (also known as NEDD4‐2), ITCH, SMURF1, SMURF2, WWP1, WWP2, NEDL1 and NEDL2.^4^ Specifically, NEDD4 consists of a C2 domain that binds phospholipids, 2–4 WW domains that identify substrates, and a catalytic domain that is homologous to the C‐terminal region of E6‐AP.^4^, ^5^ NEDD4 is highly evolutionarily conserved across multiple species and extensively expressed in mammalian tissues and has been shown to have several major cellular functions.^5^ Zhao et al. proposed that NEDD4L, a prominent member of this ligase family, exerts anti‐inflammatory effects by regulating lysophosphatidic acid receptor 1 (LPA1) signalling.^6^, ^7^ Indeed, the conditional knockout of *NEDD4L* in lung epithelial cells resulted in the development of extensive pulmonary inflammation in mature rats.^8^ Additionally, it was suggested that lipopolysaccharide possibly represses pyruvate kinase 2 (PKM2) ubiquitination by downregulating ubiquitin ligases, including NEDD4 and NEDD4L.^9^ Moreover, reactive oxygen species were shown to induce the upregulation of NEDD4 expression in primary rat cortical neurons after zinc treatment, whereas neuron pretreatment with antioxidants repressed this zinc‐induced effect.^5^ Therefore, we hypothesized that NEDD4 is involved in inflammatory responses in the lung.

In this study, a rat model of phosgene‐induced ALI^10^ was used to determine the role that NEDD4 plays in response to this potentially life‐threatening condition. Through the injection of NEDD4‐overexpressing lentiviruses into rats, we observed that the overexpression of this E3 ubiquitin ligase attenuated phosgene‐induced pulmonary injury. Furthermore, NEDD4 overexpression reduced phosgene‐induced oxidative stress and apoptosis in alveolar type II epithelial cells (AEC2s). Finally, we demonstrated that the protective effect of NEDD4 in alleviating ALI is likely mediated through its suppression of the Notch1 signalling pathway.

## 2 MATERIALS AND METHODS

### 2.1 Rat model of phosgene‐induced ALI

All the procedures involving experimental animals were approved by the Institutional Animal Care and Use Committee of Fudan University, China. Sprague–Dawley rats (6 weeks old, weighing 180–220 g) were housed in cages under a 12/12 h light/dark cycle in a room with 55%–60% humidity and a temperature of 24–26°C. The rat model of phosgene‐induced ALI was set up using a previously described method.^10^ The rats were randomly divided into four groups as follows: control (air+phosphate‐buffered saline (PBS), *n* = 6), phosgene‐exposed (phosgene +PBS, *n* = 6), phosgene/Vector (phosgene+empty vector, *n* = 6) and phosgene/NEDD4 (phosgene+NEDD4 overexpression vector, *n* = 6). The control rats were exposed to regular ambient air, whereas the other three groups were exposed to air containing phosgene (8.33 mg/L) for 5 min. The rats in phosgene/Vector and phosgene/NEDD4 groups were administered with recombinant lentivirus (1 × 10^9^ TU/ml, 100 μl per 100 g body weight) via tail vein injection at 1 h after phosgene exposure.

### 2.2 Wet‐to‐dry lung weight ratio

At 73 h after phosgene exposure and lentivirus vector injection, the animals were euthanized, and their lungs were extracted, sucked dry and weighed as the wet weight. Then, the organ was baked in an oven at 65°C for 48 h until a constant weight was obtained as the dry weight. The wet/dry lung weight ratio was calculated by dividing the wet weight by the dry weight.

### 2.3 Lentivirus construction for rat transgenesis

The *NEDD4* sequence was amplified from RNA and cloned into the pCDH‐CMV‐MCS‐EF1‐copGFP lentiviral vector to generate the pCDH‐NEDD4 transfer vector. HEK293T cells were then transfected with pCDH‐NEDD4 and the packaging plasmids psPAX2 and pMD, with the help of the Lipofectamine 2000 reagent (Invitrogen), and the packaged lentiviral particles were harvested 48 h later.

### 2.4 Plasmid construction and cell transfection

The polymerase chain reaction (PCR) was performed for amplification of the full‐length *NEDD4* cDNA. Then, the *NEDD4* cDNA was cloned into the pcDNA3.1 vector, which was subsequently transfected into alveolar type II epithelial cells (AEC2s) using Lipofectamine 2000 (Invitrogen) according to the manufacturer's instructions. The empty vector was used to set up a control.

### 2.5 Quantitative reverse‐transcription polymerase chain reaction

Total RNA was extracted from the cells using the TRIzol reagent (Invitrogen) and then reverse transcribed to cDNA with the Transcriptor First‐Strand cDNA Synthesis Kit (Roche Diagnostics Corporation). The cDNA was then amplified using the SYBR^®^ Premix Ex Taq™ II Kit (Takara), with the PCR conducted on a CFX96 Real‐Time PCR Detection System (Bio‐Rad). The following primers were used in this study: NEDD4 forward, 5′‐GGACGAGGTATGGGAGTTCT‐3′; NEDD4 reverse, 5‐CTCCACTCATCGGGTCATAC‐3′; GAPDH forward, 5′‐CAAGAAGGTGGTGAAGCAGG‐3′; and GAPDH reverse, 5′‐ CCACCCTGTTGCTGTAGCC‐3′. The relative expression level of *NEDD4* was normalized to that of glyceraldehyde 3‐phosphate dehydrogenase (*GAPDH*, the internal control), with the fold difference estimated using the 2^−ΔΔCt^ method.

### 2.6 Western blot assay

Cells or tissue extracts were prepared using RIPA lysis buffer containing a protease inhibitor cocktail solution. The cell lysates were then subjected to sodium dodecyl sulphate‐polyacrylamide gel electrophoresis, following which the separated proteins were transferred to polyvinylidene fluoride membranes (Millipore). After blocking, the membranes were incubated overnight with primary antibodies raised against the following target proteins: NEDD4 (Proteintech), neurogenic locus notch homolog protein 1 (Notch1; Cell Signaling Technology), apoptosis regulator Bax (Bax; Proteintech), Bcl‐2‐associated agonist of cell death (Bcl‐2; Proteintech), cleaved caspase‐3 (Cell Signaling Technology) and β‐actin (Proteintech). The membranes were then washed three times with Tris‐buffered saline containing 0.1% Tween 20 (TBST) and subsequently incubated with horseradish peroxidase‐conjugated secondary antibodies for 1 h. Finally, after three washes with TBST, the protein bands were detected using an enhanced chemiluminescence kit (Santa Cruz Biotechnology).

### 2.7 Lung histopathological examination

The lungs were excised from the rats in all four groups, fixed with 10% formalin, and embedded in paraffin. Then, tissue sections of 5 μm thickness were obtained and stained with haematoxylin and eosin (H&E). The severity of lung injury based on the extents of alveolar haemorrhage, oedema and inflammatory cell infiltration in bronchoalveolar lavage fluid (BALF) was expressed as a Smith score as follows: 0, no injury; 1, 1%–25% injury; 2, 25%–50% injury; 3, 50%–75% injury; and 4, 75%–100% injury.

### 2.8 Immunohistochemistry

Lung tissue samples were fixed with 4% paraformaldehyde and embedded in paraffin, following which 4‐μm‐thick sections were obtained. After their deparaffinization and rehydration, the tissue sections were stained with a primary antibody against NEDD4 (Proteintech).

### 2.9 Quantification of inflammatory factors

The rats were euthanized, and a midline thoracotomy was performed to open the rib cage. BALF was collected by flushing the lungs three times with ice‐cold PBS (1 ml each time) and then centrifuged at 600 *g* for 5 min. The BALF levels of inflammatory cytokines (tumor necrosis factor‐alpha [TNF‐α assay, Proteintech], interleukin‐6 [IL‐6 assay, Abcam], IL‐1β [assay from Abcam] and IL‐10 [assay from Proteintech]) were measured using enzyme‐linked immunosorbent assay (ELISA) kits according to the manufacturers' instructions.

### 2.10 Immunofluorescence staining

Immunofluorescence staining was performed on 4‐μm‐thick slices of paraffin‐embedded lung parenchymal tissue. In brief, after blocking the tissue sections, they were immediately incubated overnight with primary antibodies against pulmonary surfactant‐associated protein C (SFTPC; Thermo Fisher Scientific). Then, the tissue sections were incubated with a fluorescein‐labelled goat anti‐rabbit IgG antibody (Abcam), and nuclear staining was performed using 4′,6‐diamidino‐2‐phenylindole (DAPI).

### 2.11 TUNEL assay

Parenchymal tissue sections on slides were first treated with DNase‐free proteinase K. Then, after rinsing, the TUNEL assay solution was added for 1 h of incubation. Following nuclear staining with DAPI, images were captured under a fluorescence microscope.

### 2.12 Oxidative stress assay

Levels of malondialdehyde (MDA, a redox product) and superoxide dismutase (SOD, an antioxidative enzyme) within the lung parenchyma were measured using their respective assay kits (Abcam).

### 2.13 Cell culture

The RLE‐6TN cell line of AEC2s was obtained from the American Type Culture Collection. The cells were cultured in Ham's F12 medium (supplemented with 10% fetal bovine serum) at 37°C under 5% CO_2_.

### 2.14 Co‐immunoprecipitation and ubiquitination assays

For the co‐immunoprecipitation assay, ice‐cold PBS was first used to wash the cells, following which they were transfected with the *NEDD4*‐carrying vector. After transfection, the cells were lysed in lysis buffer supplemented with phosphatase and protease inhibitors. Next, the cell lysates were incubated first with the primary antibodies and then with immunoprecipitation IgG beads. The immunoprecipitates were washed with lysis buffer and boiled for 5 min in protein loading buffer. Finally, the co‐immunoprecipitated proteins were identified by Western blot assay using anti‐NEDD4 (Proteintech) and anti‐Notch1 (Proteintech) primary antibodies. For the ubiquitination assay, HA‐ubiquitin and S‐Tag‐Notch1 were added to cells that had been transfected with either the *NEDD4*‐carrying vector or the empty vector. The cells were then treated with 10 μM MG‐132 for 48 h before harvesting. The harvested cells were then subjected to immunoprecipitation with S‐Tag‐beads and immunoblotting as described above, with anti‐ubiquitin (Santa Cruz Biotechnology) and anti‐β‐actin (Proteintech) used as the primary antibodies for the Western blot assay.

### 2.15 Statistical analysis

All data are presented as the mean ± standard deviation and were analysed using GraphPad Prism 5 (GraphPad Software). Differences between groups were analysed using either Student's *t*‐test or one‐way analysis of variance, with statistical significance set at a *p* value of less than 0.05.

## 3 RESULTS

### 3.1 NEDD4 expression is downregulated in the lung tissue of phosgene‐exposed rats

**FIGURE 1:**
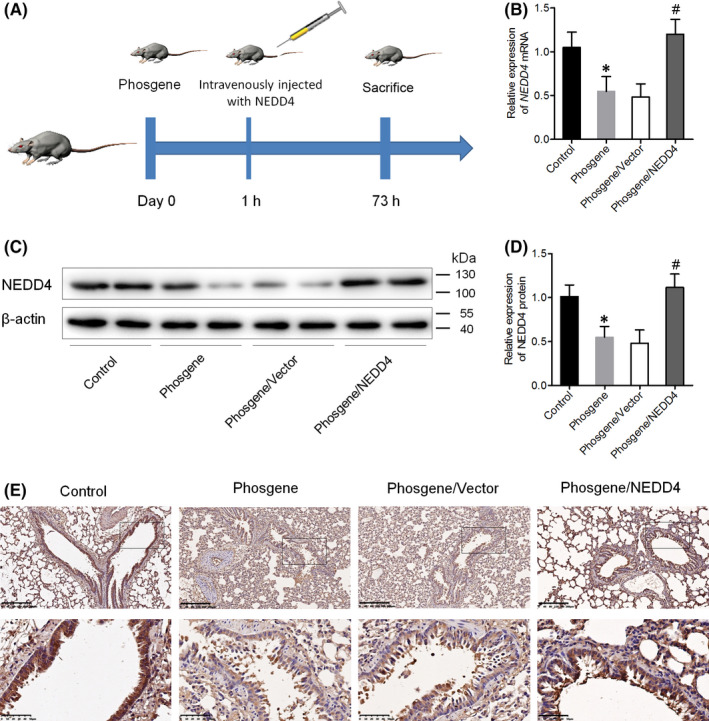
NEDD4 expression is downregulated in the lung tissue of phosgene‐exposed rats. (A) Outline of the experimental protocol. (B–D) mRNA and protein expression levels of NEDD4 in lung tissues from the different groups, measured using qPCR and western blot assays (*n* = 6 per group). (E) Immunohistochemical staining of lung tissues with anti‐NEDD4 antibody (*n* = 6 per group). Upper panel, Scale bar = 200 μm; Lower panel, Scale bar = 50 μm. *p < 0.05 vs. the control group; ^#^ *p* < 0.05 vs. the phosgene/Vector group

In a previous study, we had ascertained that phosgene exposure causes pulmonary injury in rats.^10^ In this study, three groups of rats were continuously exposed to phosgene for 5 min. For the phosgene/NEDD4 group, the animals were intravenously injected with NEDD4‐overexpressing lentiviruses at 1 h after phosgene exposure. The rats were euthanized after 73 h, and lung samples were collected for ALI evaluation, biochemical assays and histopathological examination. An outline of the experimental protocol is shown in Figure 1A. The mRNA and protein expression levels of NEDD4 were determined using quantitative PCR and Western blot assays, respectively. Compared with the NEDD4 expression levels in the lung parenchyma of rats in the control group, the levels in the phosgene‐exposed group were found to be downregulated, whereas they were upregulated in the phosgene/NEDD4 group (Figure 1B–D). These findings were corroborated by the immunohistochemical results (Figure 1E). Taken together, the results indicate that NEDD4 is downregulated in the lung tissues of phosgene‐induced rats.

### 3.2 NEDD4 overexpression attenuates phosgene‐induced inflammatory changes in lungs

**FIGURE 2:**
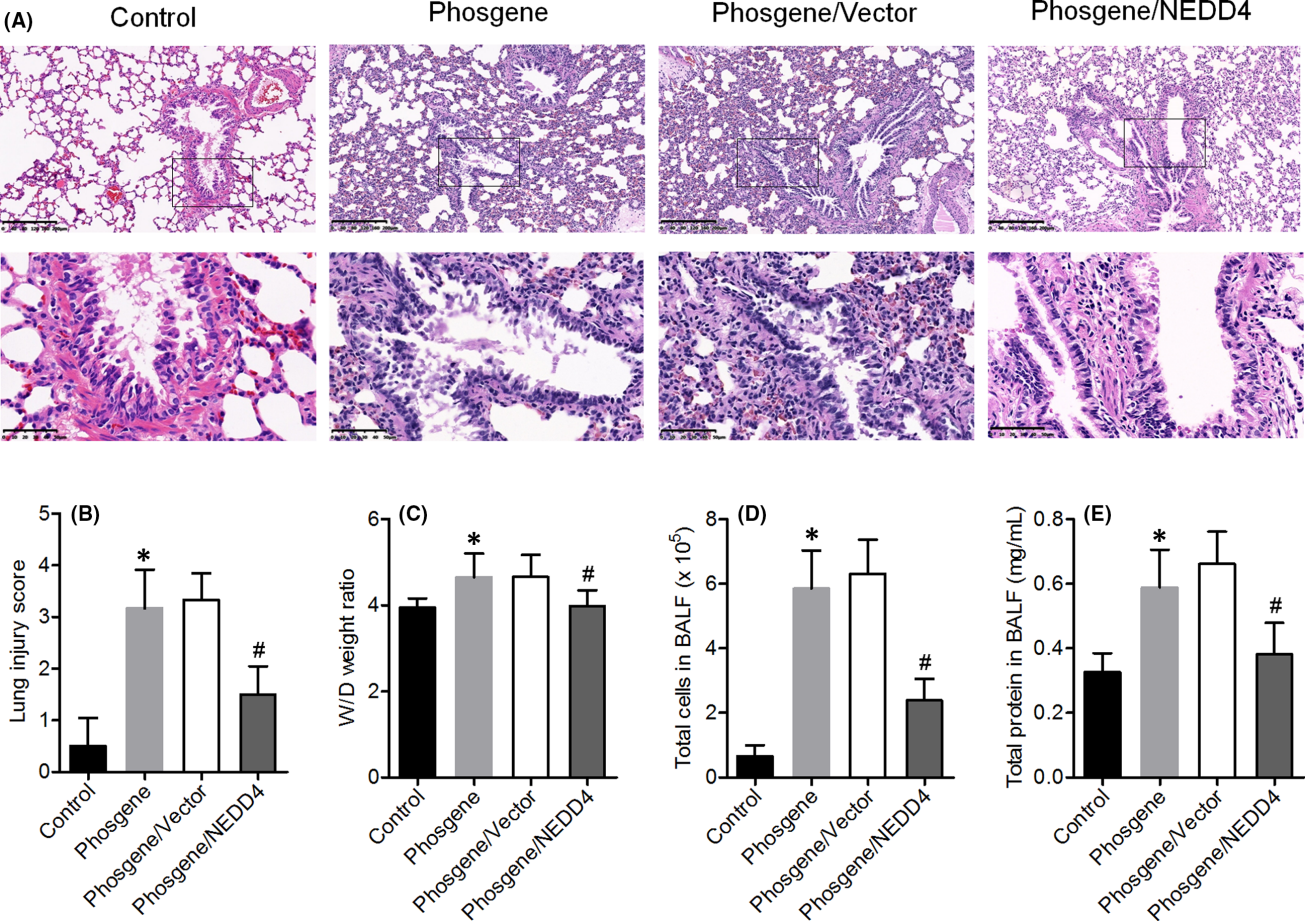
Upregulation of NEDD4 expression reduced phosgene‐induced lung inflammation. (A) H&E staining of lung tissues from rats of all groups was performed at 73 h after phosgene exposure (*n* = 6 per group). Upper panel, Scale bar = 200 μm; Lower panel, Scale bar = 50 μm. (B) Pathological scores of lung injury in the various groups (*n* = 6 per group). (C) Calculated wet/dry (W/D) lung weight ratios (*n* = 6 per group). (D) Total number of cells in BALF, quantified manually using a haemocytometer (*n* = 6 per group). (E) Total protein concentration in BALF, measured using a rat protein‐specific ELISA (*n* = 6 per group). ^#^ *p* < 0.05 vs. the phosgene/Vector group

To validate the protective effect of NEDD4 against phosgene‐induced ALI, a previously developed^10^ rat model of phosgene‐induced ALI was subjected to lentivirus‐driven NEDD4 overexpression. H&E staining of the lung parenchyma from the phosgene‐exposed rats revealed impairment of the alveolar integrity, with the noticeable extents of haemorrhage, inflammatory cell infiltration and oedema were observed. By contrast, in the phosgene/NEDD4 group, these histopathological changes were attenuated, and the Smith scores were significantly reduced (Figure 2A,B). We determined the degree of phosgene‐induced oedema by measuring the wet/dry weight ratio of the lungs. The degree of oedema was higher in the phosgene‐exposed rats than in the control rats, but this effect was attenuated in the NEDD4‐overexpressing animals (Figure 2C). To determine whether the phosgene exposure elicited an inflammatory response in rats, the total number of inflammatory cells and total amount of protein in BALF were determined. Both parameters were significantly higher in the phosgene‐exposed rats than in the control rats, but these changes were strongly attenuated in the NEDD4‐overexpressing rats (Figure 2D,E).

**FIGURE 3:**
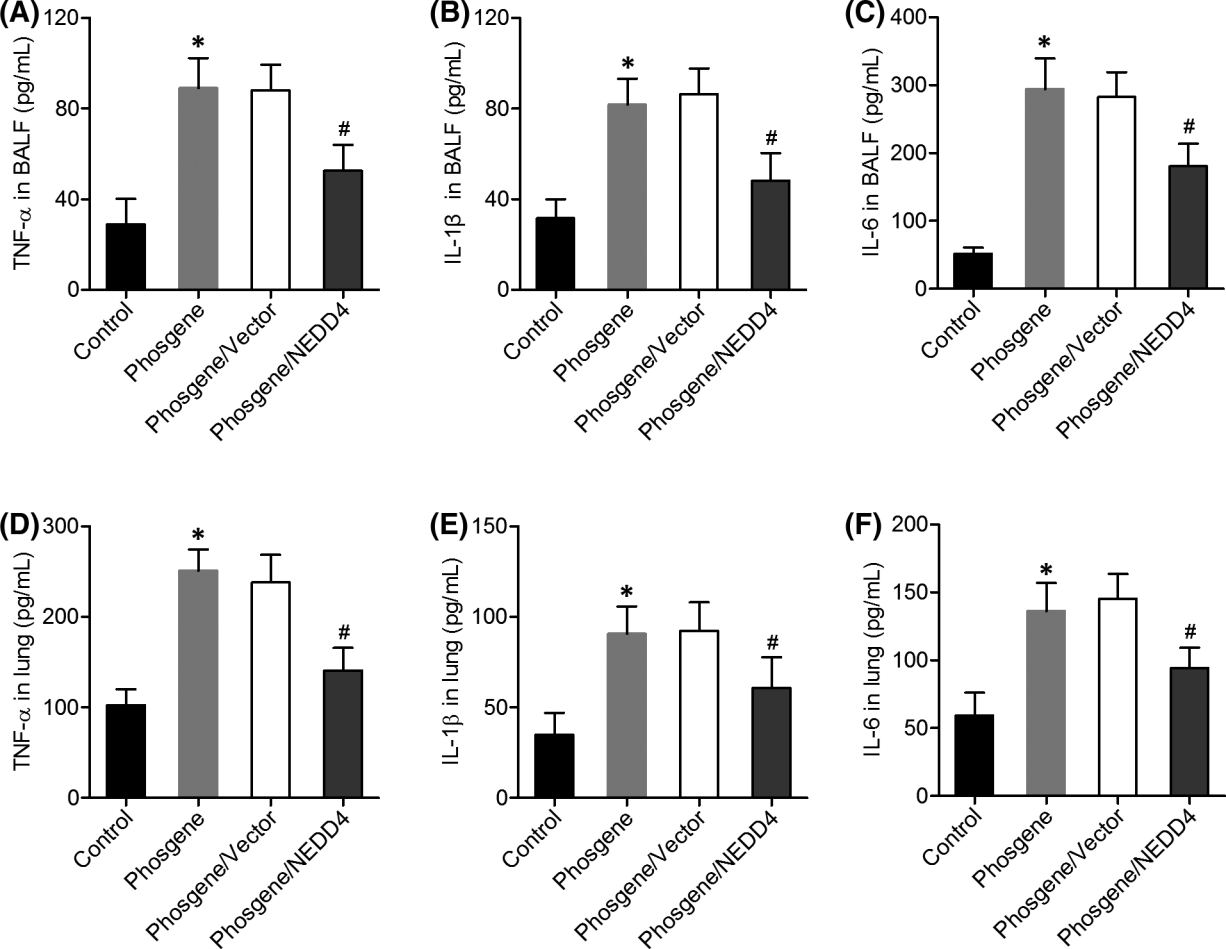
Upregulation of NEDD4 expression reduced phosgene‐induced inflammatory cytokine production. (A–C) BALF levels of secreted TNF‐α, IL‐1β, and IL‐6 (*n* = 6 per group). (D–F) Lung tissue levels of secreted TNF‐α, IL‐1β, and IL‐6 (*n* = 6 per group). *p < 0.05 vs. the control group; ^#^ *p* < 0.05 vs. the phosgene/Vector group

Additionally, we determined the levels of proinflammatory factors in the BALF and lung tissues. The ELISA results indicated that the phosgene exposure had upregulated the levels of TNF‐α, IL‐1β and IL‐6, whereas the NEDD4 overexpression weakened these alterations (Figure 3A–F). Taken together, these data suggest that overexpression of NEDD4 can alleviate the inflammatory response to phosgene exposure.

### 3.3 NEDD4 overexpression attenuates oxidative stress and promotes AEC2 survival in the lungs of phosgene‐injured rats

**FIGURE 4:**
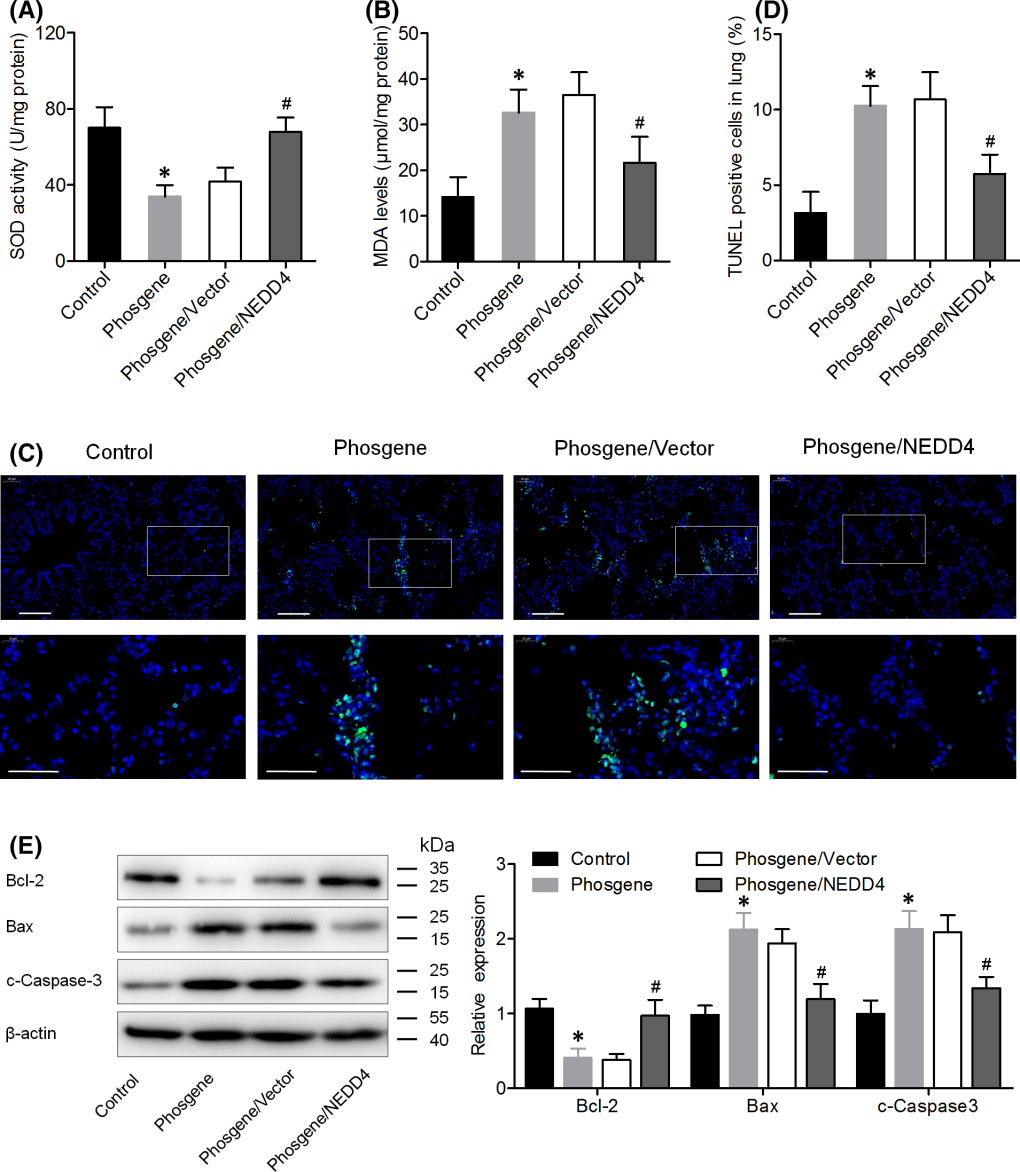
NEDD4 attenuated oxidative stress and cell apoptosis in the lung tissue of phosgene‐injured rats. (A) SOD activity and (B) MDA concentration in the lung tissue, as determined by chemiluminescence analysis (*n* = 6 per group). (C, D) Apoptosis of AEC2s in the different groups, as detected using the TUNEL assay. Quantitative analysis of TUNEL‐positive cells (*n* = 6 per group). Upper panel, Scale bar = 100 μm; Lower panel, Scale bar = 50 μm. (E) Protein levels of Bcl‐2, Bax, and cleaved caspase‐3 in the rat lungs, as determined by western blot assay (*n* = 6 per group). *p < 0.05 vs. the control group; ^#^ *p* < 0.05 vs. the phosgene/Vector group

**FIGURE 5:**
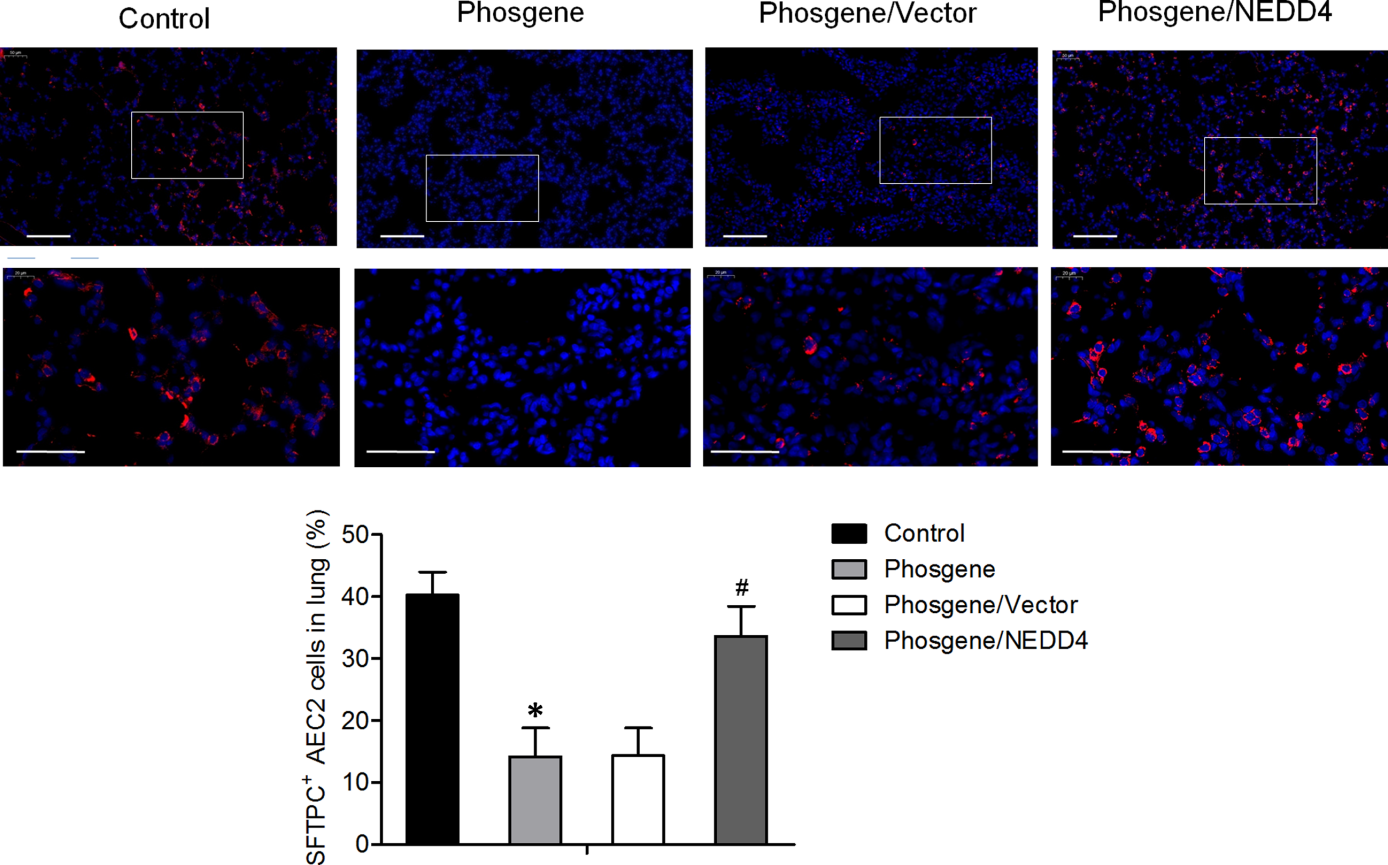
NEDD4 attenuated the change in AEC2 count in the phosgene‐injured rats. SFTPC^+^ AEC2s in the lung tissue, as detected by immunofluorescence staining (*n* = 6 per group). Quantitative analysis of the proportion of SFTPC^+^ AEC2 cells in the lung tissue. Upper panel, Scale bar = 100 μm; Lower panel, Scale bar = 50 μm

We further examined the impact of NEDD4 overexpression on the phosgene‐induced oxidative stress and survival of AEC2s. After exposure to phosgene, the activity of the antioxidative enzyme SOD was significantly decreased, and the level of MDA was increased in the lungs, but NEDD4 overexpression significantly mitigated these changes (Figure 4A,B). TUNEL results showed that the percentage of apoptotic lung cells was higher in the phosgene‐exposed rats than in the control rats, whereas it was intermediate between these two groups in the phosgene/NEDD4 group (Figure 4C,D). Additionally, in the lung parenchyma, we observed a lower level of Bcl‐2 expression and higher levels of Bax and cleaved caspase‐3 expression in the phosgene‐exposed rats than in the control rats. By contrast, these alterations proved to be attenuated in the phosgene/NEDD4 group (Figure 4E). Moreover, there was a decrease in the number of SFTPC^+^ AEC2s in the lungs of the phosgene‐exposed rats, whereas the number was increased in the NEDD4‐overexpressing animals (Figure 5). Taken together, these data reveal that NEDD4 can attenuate oxidative stress in the lungs and increase the AEC2 count in phosgene‐injured rats.

### 3.4 NEDD4 interacts with Notch1 and enhances its ubiquitination and degradation

**FIGURE 6:**
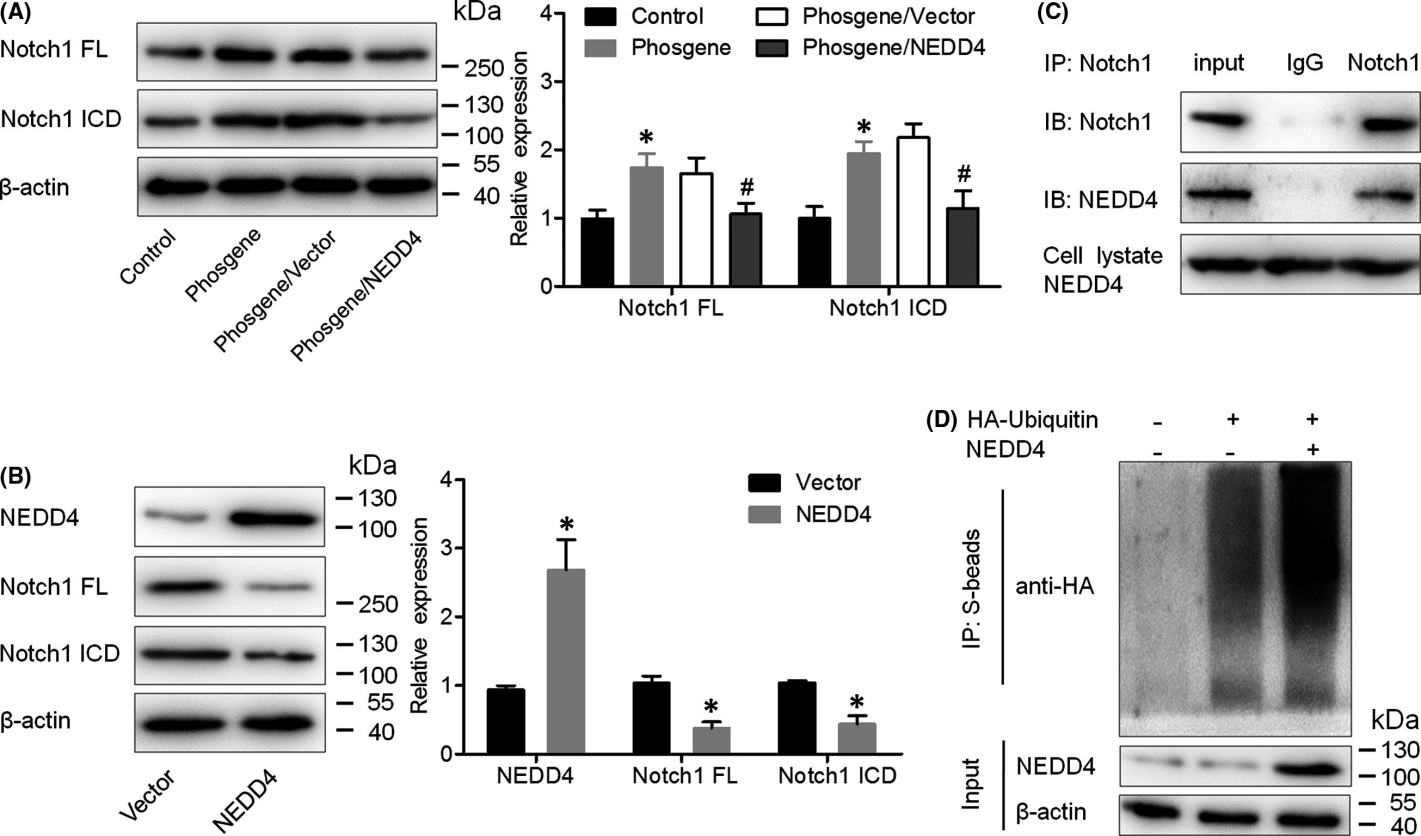
NEDD4 promoted the ubiquitination of Notch1. (A) Protein levels of Notch1 FL and Notch1 ICD in lung tissues from different groups of rats, as measured by western blot assay (*n* = 6 per group). (B) Protein levels of Notch1 FL and Notch1 ICD in AEC2s transfected with the *NEDD4*‐carrying vector or the empty vector (*n* = 3 per group). (C) Endogenous Notch1 and NEDD4 form a protein complex in AEC2s, as evidenced by co‐immunoprecipitation assay. (D) Haemagglutinin‐tagged ubiquitin was added to AEC2s transfected with either the *NEDD4*‐carrying vector or the empty vector. Cells were treated with 10 μM MG‐132 for 8 h before harvesting. The cell lysates were subjected to immunoprecipitation and immunoblotting assays as indicated (*n* = 3 per group). *p < 0.05 vs. the control group; ^#^ *p* < 0.05 vs. the phosgene/Vector group. Notch1 FL, full‐length Notch1; Notch1 ICD, cleaved activated intracellular domain of Notch1

The Notch1 signalling pathway has been implicated in lung inflammatory responses.^11^, ^12^ To elucidate the mechanism by which NEDD4 attenuates phosgene‐induced inflammatory changes, we examined its effects on Notch1 expression in phosgene‐exposed rats. According to the Western blot results, the protein levels of both full‐length Notch1 (Notch1 FL) and its cleaved activated intracellular domain (Notch1 ICD) were higher in the lung tissue of phosgene‐exposed rats than in that of the control rats, whereas they were intermediate between these two groups in rats from the phosgene/NEDD4 group (Figure 6A). Additionally, transfection of the pcDNA3.1‐NEDD4 vector into RLE‐6TN cells sharply reduced the expression levels of Notch1 FL and Notch1 ICD (Figure 6B). To further determine whether NEDD4 mediates the reduction in Notch1 levels by affecting its ubiquitination and subsequent degradation, we first tested whether it interacts with Notch1. The co‐immunoprecipitation results confirmed that NEDD4 could bind to Notch1 in RLE‐6TN cells (Figure 6C). Next, we conducted a ubiquitination assay by adding haemagglutinin‐tagged ubiquitin to AEC2s that had been transfected with either pcDNA3.1‐NEDD4 or the empty vector. Our findings indicated that the ectopic expression of NEDD4 significantly increased the amount of ubiquitinated Notch1 (Figure 6D), proving that NEDD4 interacts with Notch1 and promotes its ubiquitination and degradation.

## 4 DISCUSSION

Short‐term exposure to phosgene induces ALI, whereas lengthy exposure leads to severe acute respiratory distress syndrome and even death.^1^ Although various supportive therapies are available, treatments targeting the retrogressive physiological injury caused by phosgene have not been validated.^13^ In this study, we found that NEDD4 not only alleviated phosgene‐induced ALI but also reduced phosgene‐induced oxidative stress and AEC2 death. Furthermore, we demonstrated that the protective effect of NEDD4 in alleviating ALI is likely mediated through its suppression of the Notch1 signalling pathway.

NEDD4 is a member of the family of E3 ubiquitin ligases (SMURF1, SMURF2, WWPl, WWP2, NEDD4, NEDDL, ITCH, NEDL1 and NEDL2) that are evolutionarily conserved among eukaryotes.^5^, ^14^ Emerging evidence suggests that NEDD4 performs oncogenic functions in tumorigenesis.^15^ NEDD4^−/−^ embryos have much less motor neurons and axons, lower skeletal muscle size and abnormal (weakened) neuromuscular junction functions and assembly.^5^ The embryonic fibroblasts in NEDD4^−/−^ mice show reduced mitogenic activity.^16^ Moreover, NEDD4 has been reported to participate in both virus budding and IGF‐1 signalling during T‐cell function.^5^ Several NEDD4 family members are associated with lung inflammation. For example, melatonin attenuates sepsis‐induced lung injury by activating the serum and glucocorticoid regulated kinase 1 (SGK1)–NEDD4L pathway.^17^ Insulin mitigates pneumonia by inhibiting NEDD4L, thereby boosting the expression of the epithelial sodium channel via the phosphatidylinositol 3‐kinase–protein kinase B (PI3K–Akt) pathway.^18^ Thus, we hypothesized that NEDD4 is also involved in the physiological response to lung injury. Our study confirmed that phosgene causes ALI and that all indexes of lung injury are improved by NEDD4 overexpression. Lung injury also induces oxidative stress and the secretion of inflammatory cytokines in the organ tissue,^19^, ^20^ as confirmed in this study. Our data clearly showed that NEDD4 overexpression attenuated oxidative stress and the inflammatory response in the lungs.

AEC2s, which are alveolar progenitor cells in the mature lung, participate in the lung recovery process.^21^ It is generally accepted that among the two types of alveolar epithelial cells, the AEC2s are the most affected by exposure to adverse stimuli, which can cause mutations in or destroy these cells, thereby initiating pulmonary injury.^22^ Although these observations suggest that AEC2s are essential in the pathogenesis of pulmonary injury, the mechanisms through which the renewal of these cells is regulated for pulmonary tissue repair remain unclear. We observed a decrease in the number of AEC2s inside the lungs of phosgene‐exposed rats compared with that in the control group, whereas the number of SFTPC^+^ AEC2s was increased in the phosgene/NEDD4 group.

Notch1, an important regulator of cell differentiation and proliferation, is also associated with the cellular processes involved in myofibroblast differentiation and its inhibition would, therefore, alleviates skin, kidney and lung fibrosis.^23^ In multiple myeloma cells, NEDD4 binds specifically to the Notch1 protein and increases its ubiquitination and degradation.^24^ Specifically, NEDD4 downregulates Notch1 by steering it toward lysosomes.^25^ Of note, Notch1, Notch2 and Notch3 are strongly overexpressed in the lung tissues of rats with ovalbumin‐induced asthma, and Notch1 inhibition by emodin treatment confers more remarkable transformations within the lung tissue than does Notch2 and Notch3 inhibition.^26^ Our study confirmed that Notch1 expression was upregulated in the phosgene‐exposed group compared with its expression in the control group, but this effect was weakened in the phosgene/NEDD4 group, indicating that the Notch1 level in phosgene‐exposed rats is downregulated by NEDD4 overexpression. Moreover, the co‐immunoprecipitation and ubiquitination assays verified that NEDD4 interacts directly with Notch1 and promotes its ubiquitination and thereby its degradation.

In summary, we found that NEDD4 attenuates phosgene‐induced pulmonary injury, oxidative stress and AEC2 death. Additionally, we demonstrated that the protective effect of NEDD4 in alleviating ALI is mediated by its interaction with Notch1 for the subsequent ubiquitination and degradation of the protein. Taken together, our findings suggest that the development of methods for increasing NEDD4 expression in cells may be helpful for the treatment of diseases involving tissue atrophy.

## CONFLICT OF INTEREST

The authors declare that they have no conflict of interest.

## AUTHOR CONTRIBUTIONS

**Yiru Shao:** Conceptualization (equal); Data curation (equal); Formal analysis (equal); Funding acquisition (equal); Investigation (equal); Methodology (equal); Software (equal); Validation (equal); Visualization (equal); Writing – original draft (equal). **Zhifeng Jiang:** Formal analysis (supporting); Investigation (supporting); Software (supporting); Validation (supporting). **Daikun He:** Formal analysis (supporting); Software (supporting). **Jie Shen:** Conceptualization (equal); Project administration (equal); Supervision (equal); Writing – review & editing (equal).

## References

[1] Lu Q , Huang S , Meng X , et al. Mechanism of phosgene‐induced acute lung injury and treatment strategy. Int J Mol Sci. 2021;22(20):10933. 34681591 10.3390/ijms222010933 PMC8535529

[2] Aggarwal S , Jilling T , Doran S , et al. Phosgene inhalation causes hemolysis and acute lung injury. Toxicol Lett. 2019;15(312):204‐213. 10.1016/j.toxlet.2019.04.019 PMC6653688 31047999

[3] Shao Y , Zhou F , He D , Zhang L , Shen J . Overexpression of CXCR7 promotes mesenchymal stem cells to repair phosgene‐induced acute lung injury in rats. Biomed Pharmacother. 2019;109:1233‐1239. 30551373 10.1016/j.biopha.2018.10.108

[4] Huang X , Chen J , Cao W , et al. The many substrates and functions of NEDD4‐1. Cell Death Dis. 2019;10(12):904. 31787758 10.1038/s41419-019-2142-8 PMC6885513

[5] Boase NA , Kumar S . NEDD4: the founding member of a family of ubiquitin‐protein ligases. Gene. 2015;557(2):113‐122. 25527121 10.1016/j.gene.2014.12.020 PMC6639052

[6] Zhao J , Wei J , Dong S , et al. Destabilization of lysophosphatidic acid receptor 1 reduces cytokine release and protects against lung injury. EBioMedicine. 2016;10:195‐203. 27448760 10.1016/j.ebiom.2016.07.020 PMC5006730

[7] Louzada RA , Corre R , Ameziane El Hassani R , et al. NADPH oxidase DUOX1 sustains TGF‐beta1 signalling and promotes lung fibrosis. Eur Respir J. 2021;57(1):1901949. 32764116 10.1183/13993003.01949-2019

[8] Duerr J , Leitz DHW , Szczygiel M , et al. Conditional deletion of Nedd4‐2 in lung epithelial cells causes progressive pulmonary fibrosis in adult mice. Nat Commun. 2020;11(1):2012. 32332792 10.1038/s41467-020-15743-6 PMC7181726

[9] Ding H , Wang JJ , Zhang XY , Yin L , Feng T . Lycium barbarum polysaccharide antagonizes LPS‐induced inflammation by altering the glycolysis and differentiation of macrophages by triggering the degradation of PKM2. Biol Pharm Bull. 2020;44(3):379‐388. 33390389 10.1248/bpb.b20-00752

[10] Shen J , Wang J , Shao Y , et al. Adenovirus‐delivered angiopoietin‐1 treatment for phosgene‐induced acute lung injury. Inhal Toxicol. 2013;25(5):272‐279. 23614728 10.3109/08958378.2013.777820

[11] Zhou M , Cui ZL , Guo XJ , et al. Blockade of notch signalling by gamma‐secretase inhibitor in lung T cells of asthmatic mice affects T cell differentiation and pulmonary inflammation. Inflammation. 2015;38(3):1281‐1288. 25586485 10.1007/s10753-014-0098-5

[12] Lee E , Kang MJ , Kim JH , et al. NOTCH1 pathway is involved in polyhexamethylene guanidine‐induced humidifier disinfectant lung injuries. Yonsei Med J. 2020;61(2):186‐191. 31997628 10.3349/ymj.2020.61.2.186 PMC6992453

[13] Grainge C , Rice P . Management of phosgene‐induced acute lung injury. Clin Toxicol. 2010;48(6):497‐508. 10.3109/15563650.2010.506877 20849339

[14] Aleidi SM , Howe V , Sharpe LJ , et al. The E3 ubiquitin ligases, HUWE1 and NEDD4‐1, are involved in the post‐translational regulation of the ABCG1 and ABCG4 lipid transporters. J Biol Chem. 2015;290(40):24604‐24613. 26296893 10.1074/jbc.M115.675579 PMC4591838

[15] Wang ZW , Hu X , Ye M , Lin M , Chu M , Shen X . NEDD4 E3 ligase: functions and mechanism in human cancer. Semin Cancer Biol. 2020;67(Pt 2):92‐101. 32171886 10.1016/j.semcancer.2020.03.006

[16] Cao XR , Lill NL , Boase N , et al. Nedd4 controls animal growth by regulating IGF‐1 signaling. Sci Signal. 2008;1(38):ra5. 18812566 10.1126/scisignal.1160940 PMC2833362

[17] Li J , Liu L , Zhou X , et al. Melatonin attenuates sepsis‐induced acute lung injury through improvement of epithelial sodium channel‐mediated alveolar fluid clearance via activation of SIRT1/SGK1/Nedd4‐2 signaling pathway. Front Pharmacol. 2020;11:590652. 33362546 10.3389/fphar.2020.590652 PMC7759566

[18] Deng W , Li CY , Tong J , Zhang W , Wang DX . Regulation of ENaC‐mediated alveolar fluid clearance by insulin via PI3K/Akt pathway in LPS‐induced acute lung injury. Respir Res. 2012;30(13):29. 10.1186/1465-9921-13-29 PMC3362779 22458687

[19] Yang L , Liu G , Fu L , Zhong W , Li X , Pan Q . DNA repair enzyme OGG1 promotes alveolar progenitor cell renewal and relieves PM2.5‐induced lung injury and fibrosis. Ecotoxicol Environ Saf. 2020;1(205):111283. 10.1016/j.ecoenv.2020.111283 32977282

[20] He DK , Shao YR , Zhang L , et al. Adenovirus‐delivered angiopoietin‐1 suppresses NF‐kappaB and p38 MAPK and attenuates inflammatory responses in phosgene‐induced acute lung injury. Inhalation Toxicol. 2014;26(3):185‐192. 10.3109/08958378.2013.872213 24517841

[21] Desai TJ , Brownfield DG , Krasnow MA . Alveolar progenitor and stem cells in lung development, renewal and cancer. Nature. 2014;507(7491):190‐194. 24499815 10.1038/nature12930 PMC4013278

[22] Yuan T , Volckaert T , Redente EF , et al. FGF10‐FGFR2B signaling generates basal cells and drives alveolar epithelial regeneration by bronchial epithelial stem cells after lung injury. Stem Cell Reports. 2019;12(5):1041‐1055. 31056475 10.1016/j.stemcr.2019.04.003 PMC6524168

[23] Hu B , Phan SH . Notch in fibrosis and as a target of anti‐fibrotic therapy. Pharmacol Res. 2016;108:57‐64. 27107790 10.1016/j.phrs.2016.04.010 PMC5074914

[24] Che F , Chen J , Wan C , Huang X . MicroRNA‐27 inhibits autophagy and promotes proliferation of multiple myeloma cells by targeting the NEDD4/Notch1 axis. Front Oncol. 2020;10:571914. 33262943 10.3389/fonc.2020.571914 PMC7686543

[25] Zhu JY , Heidersbach A , Kathiriya IS , et al. The E3 ubiquitin ligase Nedd4/Nedd4L is directly regulated by microRNA 1. Development. 2017;144(5):866‐875. 28246214 10.1242/dev.140368 PMC5374346

[26] Hua S , Liu F , Wang M . Emodin alleviates the airway inflammation of cough variant asthma in mice by regulating the notch pathway. Med Sci Monit. 2019;29(25):5621‐5629. 10.12659/MSM.915080 PMC6685324 31354164

